# Complete sequence cloning and bioinformatics analysis of the chukar partridge (*Alectoris chukar*, Aves, Galliformes) in Guangxi base on mitochondrial genome

**DOI:** 10.1080/23802359.2017.1339215

**Published:** 2017-06-16

**Authors:** Yanfang Zhang, Zhixun Xie, Xianwen Deng, Zhiqin Xie, Liji Xie, Qing Fan, Sisi Luo, Jiabo Liu, Li Huang, Jiaoling Huang, Tingting Zeng, Sheng Wang

**Affiliations:** Department of Biotechnology, Guangxi Key Laboratory of Veterinary Biotechnology, Guangxi Veterinary Research Institute, Nanning, China

**Keywords:** Mitochondrial genome, chukar partridge, *Alectoris chukar*, genome organization

## Abstract

The objective of this study was to obtain the complete mitochondrial DNA sequence of chukar partridge, and to provide reference data for protection and utilization of these resources of chukar partridge. The complete mitochondrial genome sequence of the China chukar partridge was measured by PCR-based methods and analysed in detail. Our research findings reveal that the entire mitochondrial genome of the chukar partridge is a circular molecule consisting of 16,688 bp (GenBank accession number: KY829450). The contents of A, T, C, and G in the mitochondrial genome were found to be 30.44%, 24.43%, 31.57%, and 13.56%, respectively. The complete mitochondrial genome of the chukar partridge has a typical structure, including 13 protein-coding genes, two rRNA genes, 22 tRNA genes, and one control region (D-loop region). This complete mitochondrial genome sequence provides essential information in understanding phylogenetic relationships among Galliformes mitochondrial genomes.

The chukar partridge (*Alectoris chukar*, Aves, Galliformes) has a very wide distribution, ranging from east Balkans and the adjacent Mediterranean islands to central Asia up to northeastern China (Cheng 1978; Aebischer 1997; Madge and McGowan 2002). The chukar is a polytypic species, with 16 reported subspecies in the world (Song and Liu 2013) and six described subspecies in China (Zheng 2005). The Chukar partridge in our report, is one of the subspecies from Guangxi, China, was classified as Least Concerned (LC) in the International Union for the Conservation of Nature and Natural Resources (IUCN) Red list (IUCN 2012). Many studies showed that mitochondrial (mt) DNA sequences are suitable markers to infer genetic diversity and phylogeny (Xie et al. 2016a; Zhang et al. 2016a, 2016b, 2016c).

Chukar partridge specimen was collected from Nanning, Guangxi province, China. The specimens were kept in the laboratory at −80 °C under the accession serial no. 60813984. In this report, we describe the mt genome of Chukar partridge genomic DNA extracted from its muscle tissue using the EasyPure Genomic DNA Kit (Beijing, China). DNA was stored at −20 °C. The DNA sequence was analysed using DNAStar 7.1 software (Madison, MI) and the base composition and distribution of the mtDNA sequence were analysed using tRNA Scan-SE1.21 (Santa Cruz, CA) (http://lowelab.ucsc.edu/tRNAscan-SE/) (Lowe and Eddy 1997) and DOGMA software (Austin, TX) (http://dogma.ccbb.utexas.edu/) (Wyman et al. 2004), respectively.

Whole mtDNA sequence of Chukar partridge has a circular genome of 16,688 bp containing 13 protein-coding genes, 22 tRNA genes, two rRNA genes, and one control region, which are similar to those of other avian species in gene arrangement and composition (Xie et al. 2016b). A, T, C, and G in the mt genome were found to be 30.44%, 24.43%, 31.57%, and 13.56%, respectively. The entire mt genome sequence has been deposited in GenBank recently (GenBank accession number: KY829450). MtDNA analysis revealed that the D-loop is a non-coding control region located at 1154 bp position between the tRNA*^Glu^* and tRNA*^Phe^* genes. Twenty-two deduced tRNA genes were found to be distributed in rRNA and protein-coding genes, ranging from 66 to 76 bp in size. The lengths of the 12S rRNA and 16S rRNA genes are 966 bp and 1617 bp, respectively.

The mt genes do not encode on the H-strand including 1 protein-coding gene (*ND6*) and eight tRNA genes, which are similar to typical avian mtDNAs (Xie et al. 2016c). The initiation codon of the protein-coding genes is ATG, except for *COX1*, which shows a GTG initiation codon. These genes have four different types of termination codons and are found in most vertebrates (David et al. 1998).

With respect to the mtDNA obtained in this study, the DNA data of 13 protein coding genes of chukar partridge and 11 other Galliformes were used to build the neighbour-joining phylogenetic tree. The NJ tree method was performed using MEGA 6.0 (Chemnitz, Germany) (http://www.megasoftware.net) with 1000 bootstrap replicates (Figure 1).

**Figure 1.**
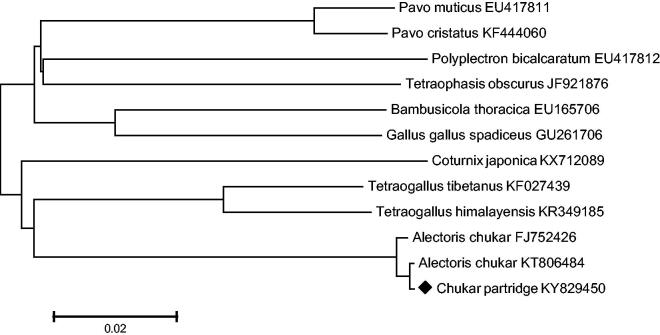
A neighbour-joining (NJ) tree of 12 species from Galliformes was constructed based on the dataset of 13 concatenated mitochondrial protein coding genes.
